# Origin of the ease of association of color names: Comparison between humans and AI

**DOI:** 10.1177/20416695221131832

**Published:** 2022-10-26

**Authors:** Hidehiko Komatsu, Ami Maeno, Eiji Watanabe

**Affiliations:** 68292 National Institute for Basic Biology, Okazaki, Aichi, Japan; Brain Science Institute, 13089 Tamagawa University, Machida, Tokyo, Japan; 12875 Kyoto Institute of Technology, Kyoto, Japan; 68292 National Institute for Basic Biology, Okazaki, Aichi, Japan; Graduate University for Advanced Studies (SOKENDAI), Okazaki, Japan

**Keywords:** color name, language, humans, AI, GPT-3

## Abstract

Rapid evolution of artificial intelligence (AI) based on deep neural networks has resulted in artificial systems such as generative pre-trained transformer 3 (GPT-3), which can generate human-like language. Such a system may provide a novel platform for studying how human perception is related to knowledge and the ability of language generation. We compared the frequency distribution of basic color terms in the answers of human subjects and GPT-3 when both were asked similar questions regarding color names associated with the letters of the alphabet. We found that GPT-3 generated basic color terms at a frequency very similar to that of human non-synaesthetes. A similar frequency was observed when color names associated with numerals were tested indicating that simple co-occurrence of alphabet and color word in the trained dataset cannot explain the results. We suggest that the proposed experimental framework using the latest AI models has the potential to explore the mechanisms of human perception.

The rapid evolution of artificial intelligence (AI) based on deep neural networks (NNs) has resulted in the development of artificial systems. These systems can generate natural texts whose source of generation (humans or AI systems) is difficult to distinguish. Generative pre-trained transformer 3 (GPT-3) (Brown et al., 2020a, 2020b) is one of the most advanced examples of such systems that can understand and generate natural language. Briefly, GPT-3 is a massive NN that inputs and outputs “tokens,” the smallest units that constitute a sentence, such as words and symbols. Given a token sequence and various control parameters, the GPT-3 predicts the next token based on the token type and the token's position in the token sequence. The predicted tokens are combined into the token sequence to generate sentences and the process is executed recursively. GPT-3 predictions were learned from approximately 300 billion tokens from the Internet text and digital archive written in English that covers large domains of human knowledge. Such a system may provide a novel platform to study how human perception is related to the knowledge and ability of language generation. This is because the responses of artificial systems such as GPT-3 are based on these two factors.

As a first attempt, in this study, we tested the responses of GPT-3 to simple questions regarding color names and examined the frequency distribution of basic color terms in the answers. The basic color terms correspond to 11 irreducible English color names from Berlin and Kay (1969): black, white, red, yellow, green, blue, brown, orange, purple, pink, and gray. When people are asked to provide color names, some colors are provided earlier and more frequently than others (Battig and Montague, 1969). Importantly, such variations in the ease of generation of basic color names correspond to neither the color word frequency in the corpus (Simner et al., 2005) nor the order of typology (Berlin and Kay, 1969; Kay and Regier, 2003), and the origin of the difference in the ease of generation across basic color terms is unclear. We speculate if this phenomenon originates from the general knowledge of humans and the ability to generate language, GPT-3 would provide basic color terms in an order comparable to that provided by humans. This problem was examined in the present study.

## Methods

**Figure 1.**
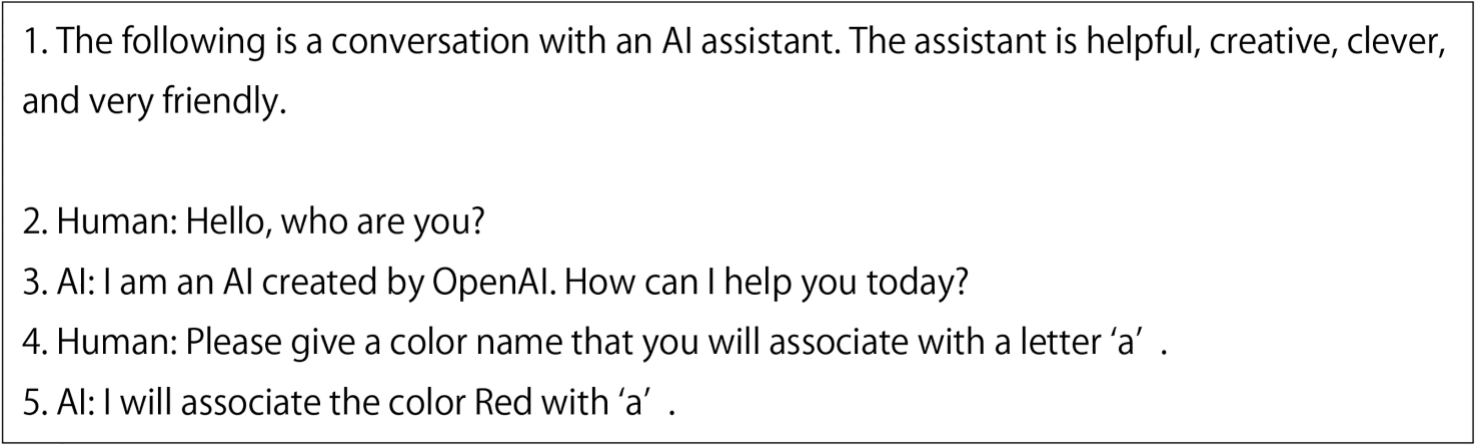
An example of the texts of question and answer with GPT-3 for the case of letter ‘a'. We used Chat function at https://beta.openai.com/examples and placed the question at the Playground. The first to the third sentences show the default texts given by GPT-3 which we did not touch. The fourth line is the question which we gave to GPT-3 for the case of letter ‘a’, and the fifth line shows an example of the answer of GPT-3. Numbers at the left are added for the purpose of explanation.

To obtain the frequency distribution of basic color terms from GPT-3, we employed a simple question-and-answer test that was employed to study the association of graphemes to colors in human subjects (Simner et al. 2005). In this study, the experimenter presented a questionnaire that asked the subjects to give any color association for the 26 letters of the alphabet. We used this procedure because in their study, it was found that the order of the frequency of basic color terms in non-synaesthetes was approximately comparable to that of the ease of generation of color terms in human subjects (Battig and Montague, 1969), but it did not correspond to the color word frequency in the corpus (Simner et al., 2005) or the order of typology (Berlin and Kay, 1969) of basic color terms. Similarly, in the present study, we asked GPT-3 to give a color name associated with each of the 26 letters of the alphabet. Figure 1 is an example of the text for the case of letter “a.” We used the Chat function at http://beta.openai.com/examples and placed the question in Playground. The first to third sentences in Figure 1A are the default texts provided by the Chat function of GPT-3. The fourth line is the question that we inputted into GPT-3 for the case of letter “a,” and the fifth line is an example of the answer of GPT-3. After recording the answers of GPT-3, we erased the fifth line. Thereafter, we inputted the next question into GPT-3. In the main experiment, we used the “Davinci” engine of GPT-3, which, although slow, outputs the most accurate and fluent texts, and tested at four “temperature” parameter values: 0.3, 0.5, 0.7, and 0.9. Temperature parameter in GPT-3 controls the randomness/variations of the model output. Other parameters of GPT-3 include top_P, freauency_penalty, and presence_penalty. Top_P parameter determines how much of the top probability of the predicted token should be targeted for output. Since top_P and temperature parameters are expected to have similar effects, only the value of temperature was varied in the present study and the top_P was untouched from the default value (1). Both frequency_penalty and presence_penalty are parameters that suppress token repetition. As the present study did not need to control the token repetition, we left those parameters untouched from the default value (0 and 0.6, respectively).

At each temperature, we repeated the question-and-answer test 50 times for each letter of the alphabet. Therefore, a total of 1,300 answers from GPT-3 were obtained. We counted the number of times each of the 11 basic color terms appeared in the answer and obtained the frequency distribution of the basic color terms. In the present study, we compared the frequency distributions obtained by GPT-3 and those reported for human subjects. We used the answer for analysis only when it specified one of the 11 basic color terms. We also tested the performance of GPT-3 using a different engine (Ada), which, although fast, has low accuracy. Regarding this supplementary test, we repeated the test 20 times for each letter at only one temperature value (0.7). We also tested the frequency distribution of basic color terms associated with Arabic numerals (0 to 9) by GPT-3 (Davinci engine at temperature 0.9) using a question-and-answer test similar to the one used for the main experiment. In this test, the color name associated with each numeral instead of alphabet was asked; e.g., for the case of “0,” the question was “Please give a color name that you will associate with a number ‘0'.” We repeated this test 40 times for each numeral. Tests using GPT-3 were conducted between September 2021 and February 2022.

## Results

**Figure 2.**
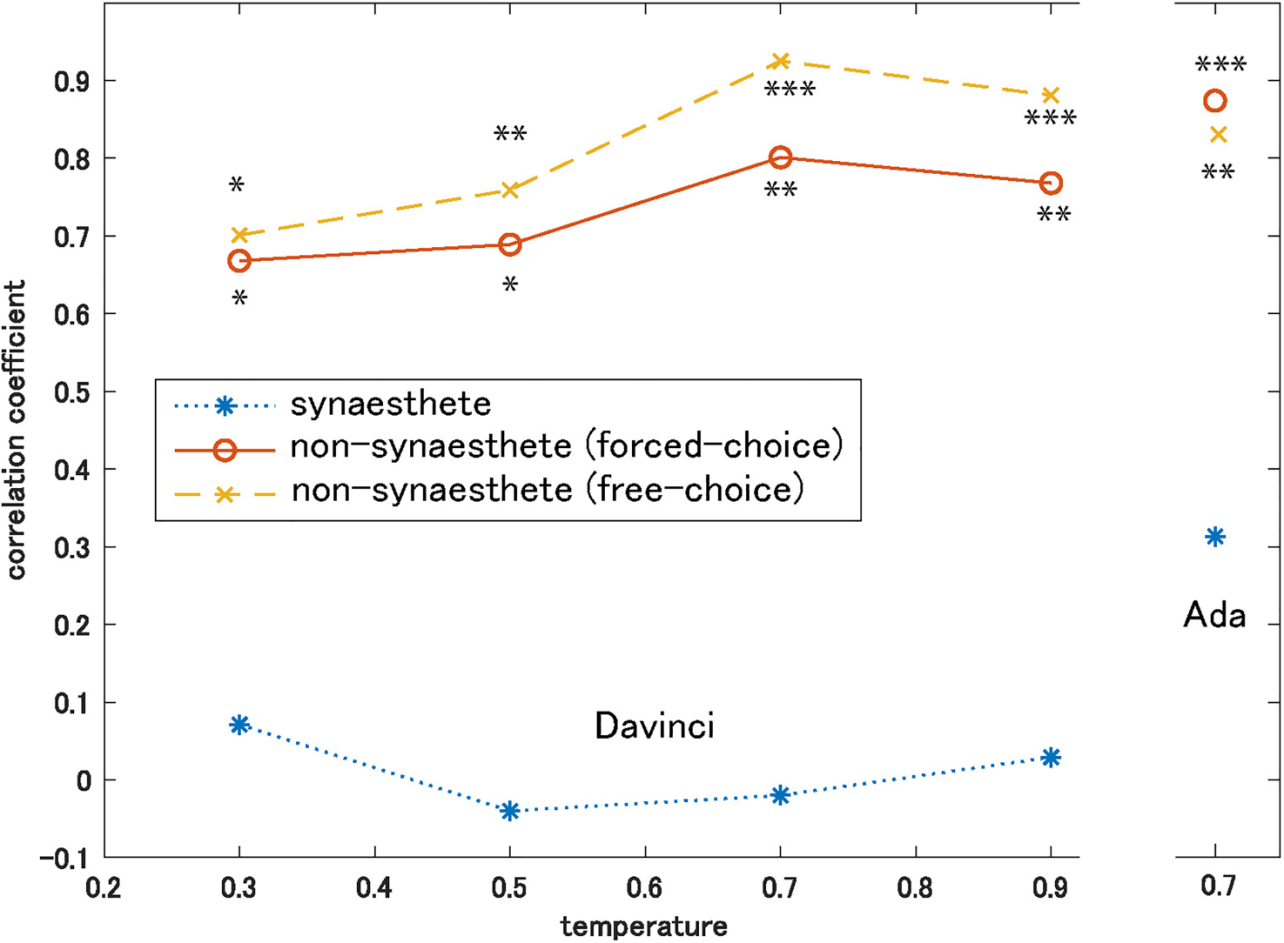
Correlation coefficients computed between the log-transformed frequencies of basic color terms given by GPT-3 and those given by human synaesthetes and non-synaesthetes. Human data were adopted from Simner et al. (2005). Left; Results obtained by Davinci engine at four temperatures of GPT-3. Right; Results obtained by Ada engine at temperature of 0.7. *p < 0.05, **p < 0.01, ***p < 0.001.

The frequency distributions of the basic color terms that appeared in the GPT-3 responses for each temperature are summarized in Table 1. Some color terms appeared more frequently than others in the answers, and there were some differences in the distribution across different temperatures. We compared the responses of GPT-3 with those of human subjects tested using a similar procedure (Simner et al., 2005). In this study, the human subjects consisted of individuals with and without grapheme-color synesthesia. Non-synaesthetes were tested under two conditions: forced- and free-choice. In the forced-choice condition, the subjects were forced to answer a color name for each alphabet, while in the free-choice condition, they were asked to note a color if one easily came to mind. Table 2 summarizes the frequency distributions of the basic color terms for the three conditions reported by Simner et al. (2005). We quantitatively evaluated the similarity of the frequency of the color names used by GPT-3 and those used by human subjects. Because the frequency of the color names was skewed, we first log-transformed the frequency value of each color. Before log-transformation, we added the minimum number of non-zero value (0.000769 that was for brown in GPT-3 at temperature 0.5) to avoid the presence of zero value (3 cases in GPT-3: gray and brown at temperature 0.3, gray at 0.5). Then, a Shapiro–Wilk test was performed for each data and none showed evidence of non-normality (*W*  =  0.93, *p*  =  .41 for GPT-3 at *t*(temperature)  =  0.3; *W*  =  0.92, *p*  =  .30 for GPT-3 at *t*  =  0.5; *W*  =  0.95, *p*  =  .60 for GPT-3 at *t*  =  0.7; *W*  =  0.95, *p*  =  .67 for GPT-3 at *t*  =  0.9; *W*  =  0.86, *p* = .0507 for Synaestetes of Simner et al. (2005); *W*  =  0.86, *p*  =  .07 for nonSynaesthetes forced choice; *W*  =  .90, *p*  =  .17 for non-Synaesthetes free choice). Based on this, we computed Pearson's correlation coefficient between the log-transformed value of the answers of GPT-3 and those of human subjects. The left side of Figure 2 shows the correlation coefficients computed at four temperatures of GPT-3 using the Davinci engine with human synaesthetes and non-synaesthetes (forced- and free-choice). It can be observed that correlation is low (<0.1) between human synaesthetes and GPT-3 (insignificant for all temperatures); in comparison, that between human non-synaesthetes and GPT-3 is higher. The correlation was significant between GPT-3 and humans (both forced- and free-choice groups) at all temperatures while it tended to increase with an increase in temperature. The significance levels are shown in Figure 2. The correlations for the free-choice group were slightly higher than those for the forced-choice group though there were no significant differences between two groups at any temperature. These results show that GPT-3 provides basic color terms in an order comparable to that of humans when the temperature is high.

**Table 1.** Frequency distribution of basic color terms in the answers of GPT-3 with the Davinci engine.

	Red	Blue	Green	Yellow	Orange	Purple	Pink	Brown	White	Black	Gray
Temp = 0.9	0.12	0.10	0.07	0.07	0.04	0.03	0.03	0.01	0.02	0.01	0.01
Temp = 0.7	0.19	0.16	0.09	0.08	0.06	0.04	0.02	0.01	0.02	0.02	0.00
Temp = 0.5	0.16	0.14	0.06	0.06	0.02	0.01	0.04	0.00	0.04	0.01	0.00
Temp = 0.3	0.48	0.19	0.08	0.07	0.02	0.00	0.04	0.00	0.04	0.00	0.00

*Note*.In some cases, GPT-3 gave a color name (e.g., cyan) that was not basic color names, or more than two color names (e.g., blue, purple, green). In other cases, GPT-3 gave a word that is different from the color name (e.g., banana) or uninterpretable words. This is the reason why the overall frequency was less than one. Temp = temperature; GPT-3 = generative pre-trained transformer 3.

**Table 2.** Frequency distribution of basic color terms in the answers of human subjects reported by Simner et al. (2005).

	Red	Blue	Green	Yellow	Orange	Purple	Pink	Brown	White	Black	Gray
Synaesthetes	0.11	0.10	0.11	0.13	0.05	0.05	0.04	0.13	0.09	0.08	0.11
Non-syn (forced)	0.13	0.14	0.14	0.14	0.07	0.07	0.04	0.07	0.08	0.08	0.04
Non-syn (free)	0.14	0.14	0.15	0.13	0.09	0.10	0.04	0.04	0.06	0.08	0.03

Synaesthetes  =  grapheme-color synaesthetes (Figure 2 of Simner et al. 2005); Non-syn (forced)  =  Forced choice by non-synaesthetes (Figure 3 of Simner et al. 2005); Non-syn (free)  =  Free choice by non-synaesthetes (Figure 4 of Simner et al. 2005).

To examine whether the ability to generate natural language affects the frequency distribution of basic color terms, we tested the performance of GPT-3 with the Ada engine at a temperature of 0.7, in which correlation was quite high for the Davinci engine. The frequency distributions of the basic color terms that appeared in the answers of the Ada engine are summarized in Table 3. As was done for the data obtained by Davinci engine, we first log-transformed the frequency values after adding the same constant value (0.000769), tested the normality of the data (*W* = 0.97, *p*  =  0.92, Shapiro–Wilk test), then computed Peason's correlation coefficient. As shown on the right side of Figure 2, the correlation coefficients between the answers of the Ada engine and human non-synaesthetes are comparable to those of the Davinci engine at the same temperature (0.7). The correlation with the human synaesthete was quite low, as observed for the Davinci engine. These results suggest that as far as the simple question-and-answer task is used, the performance of GPT-3 does not clearly depend on the engine employed.

**Table 3.** Frequency distribution of basic color terms in the answers of GPT-3 with the Ada engine.

	Red	Blue	Green	Yellow	Orange	Purple	Pink	Brown	White	Black	Gray
Temp = 0.7	0.09	0.32	0.17	0.01	0.01	0.01	0.00	0.00	0.02	0.04	0.00

Temp = temperature; GPT-3 = generative pre-trained transformer 3.

We verified whether the high correlation in performance between the GPT-3 and human non-synaesthetes observed in the present study is specific to English speakers and the Roman alphabet. Simner et al. (2005) conducted the same test on German non-synaesthetic speakers. The correlation between German and English speakers (forced-choice non-synaesthetes) was quite high (*r*  =  0.811), whereas that for English speakers (free-choice non-synaesthetes) was not as high (*r*  =  0.617). Similarly, the performance of GPT-3 in the present study was not highly correlated with the results of the German subjects (*r*  =  0.413–0.461). Nagai et al. (2016) examined color associations with graphemes in a non-synaesthetic Japanese population. The frequency distributions of basic color terms associated with graphemes (kana characters, alphabets, and Arabic, and kanji numerals) are shown in Figures S2 and S3 of their study. The same test was conducted twice, and the results of the two tests were similar. When we computed the correlation coefficient between the frequency distribution of their results (average of first and second tests) and the performance of GPT-3 (Davinci engine), we found that the correlation was quite high for all graphemes (*r*  =  0.974 and 0.979; for kana character, *r*  =  0.907 and 0.934; for alphabets, *r*  =  0.887 and 0.929; for Arabic numerals, *r*  =  0.889 and 0.900; for kanji numerals, *r*  =  0.861 and 0.798; temperature  =  0.7 and 0.9, respectively). We also tested the frequency distribution of basic color terms associated with Arabic numerals (0–9) by GPT-3 (Davinci engine at temperature 0.9) (see “Methods” section), and the results (Table 4) were compared with the frequency distribution of the basic color terms associated with alphabet by GPT-3 of the same condition. We found that the correlation between the two results was quite high (*r*  =  0.972 and 0.963 with the Davinci engine; temperature  =  0.7 and 0.9, respectively). In these additional analyses, again, we first log-transformed the frequency values after adding the same constant value (0.000769), tested the normality of the data (*p* > .05, Shapiro–Wilk test), then computed Pearson's correlation coefficient. These results suggest that the frequency distribution of color names generated by GPT-3 is not specifically related to a certain language (e.g., English) nor to a certain index (e.g., alphabet), although there are variations in the performance of human subjects owing to unknown factors.

**Table 4.** Frequency distribution of basic color terms in the answers of GPT-3 with the Davinci engine when the association with numerals were tested.

	Red	Blue	Green	Yellow	Orange	Purple	Pink	Brown	White	Black	Gray
Temp = 0.9	0.17	0.09	0.06	0.06	0.06	0.04	0.02	0.01	0.02	0.03	0.01

Temp = temperature; GPT-3 = generative pre-trained transformer 3.

## Discussion

In this study, using a procedure analogous to that used for human subjects employing natural language questions, we observed that GPT-3 can generate basic color terms at a frequency very similar to that of human non-synaesthetes. The similarity was more distinct when GPT-3 allowed a larger degree of variability (high temperature). Presumably, an increase in the temperature value increased the likelihood that minor color names weekly associated with the letter to appear. Importantly, we did not ask GPT-3 to answer the frequency of color names. We simply asked for the color name associated with each alphabet, and the frequency of color names was indirectly evaluated from the statistics of the answers. It is highly unlikely that the present results can be explained by simple co-occurrence of alphabet and color names in the trained data of GPT-3 because similar results were obtained when numerals instead of alphabets were used. We also directly examined this problem by Ngram analysis (bigram, trigram, 5gram, and 10gram) and unigram analysis using a large-scale dataset from WikiText-103 that contains over 100 million tokens (Supplemental Tables S1 and S2). We found that the co-occurrence probability was very similar for all cases tested, and it was highly correlated with the unigram computed by the basic color terms (Supplemental Table S3). On the other hand, these co-occurrence probabilities were quite different from the frequency distribution of the basic color terms generated by GPT-3 in response to either the alphabets or the numerals. The correlations between the frequency distribution of the color terms generated by GPT-3 and the co-occurrence probability between the alphabets/numerals and color terms are very low (Supplemental Table S4). These results support our assumption that the present results cannot be explained by simple co-occurrence of alphabet and color names in the trained data of GPT-3.

For the human subjects, the determinant of the order of the frequency of the generation of color names is not completely understood. In the study by Simner et al. (2005), the order of the frequency of basic color terms in non-synaesthetes was approximately comparable to that of the ease of generation of color terms in human subjects (Battig and Montague, 1969). However, it did not correspond to the color word frequency in the corpus (Simner et al., 2005) or the order of typology (Berlin and Kay, 1969) of basic color terms. Because the results of the present study are highly correlated with those of Simner et al. (2005), the order of frequency of color-term generation by GPT-3 corresponds to the ease of generation of color terms in human subjects. However, it does not correspond to the frequency in the corpus or the order of typology. The ease of generation is related to the exemplar typicality (Simner et al. 2005) and we consider this should be related to the structure of general knowledge humans have of the world. GPT-3 is trained with huge amount of text data present in the web and digital archive which is not restricted to the knowledge of a specific domain but is related to every aspect of knowledge related to the natural and artificial world (general knowledge). GPT-3 is highly capable of handling natural language which is useful to extract meaningful information from the text dataset. In addition, ease of generation is directly related to the function of lexical retrieval. Therefore, we speculate that the similarity in the generation of basic color terms between human subjects and GPT-3 stems from the general knowledge and language ability that is shared by human subjects and GPT-3. A high correlation between the human association of basic color terms with numerals is in line with this interpretation. However, how the natural language ability of GPT-3 is related to the acquisition of the ease of generation is still an open question. To examine whether AI without natural language ability can acquire the human-like ease-of-generation ordering of colors may be useful to answer this question in future experiments.

So far, we have discussed on the cause of the similarity of the frequency distribution of the basic color terms generated by GPT-3 and human non-synaestheses considering only the summary statistics of the frequency of color names. However, this problem can be considered from another perspective taking into account how the color names were associated with each alphabet or each numeral. Because the sum of the frequency distribution of all combinations of the alphabet and the color name (e.g., “a/red,” “b/red,” “c/red,” … “a/blue,” “b/blue,” …) should yield the summary statistics, this measure may also provide useful information when considering the mechanism of the generation of the frequency distribution of color terms. The present experiment employed a question-and-answer test for each letter, which was very similar to that reported by Simner et al. (2005). They analyzed the effects of various factors on the generation of color terms for each alphabet and observed that the initial letter of the color terms (e.g., “r” for red) tended to be associated with the corresponding color terms in both the forced- and free-choice groups of non-synaesthetes, although the effects could explain only a small part of the entire frequency distribution. We also observed that GPT-3 exhibits a tendency of initial-letter match (e.g., 16 cases of blue for “b,” 20 cases of red for “r” at a temperature of 0.9 for the Davinci engine); nonetheless, the removal of this effect did not affect the entire result (data not shown). In a more recent paper, Mankin and Simner (2017) showed that letter-color association in non-synaestheses (as well as synaestheses) is influenced by the letter-word association (e.g., apple for A) and color-word association (e.g., red for apple). This suggests that letter-color association is mediated by two separate associations: one is the association of prototypical word for a particular letter (e.g., apple for A), and the other is the association of prototypical color of the above word (e.g., red for apple). Both of these associations should be part of the general knowledge of English speaking population. Their paper suggests a potential mechanism that connect letter and color name. Although their results can explain only a part of the specific association between alphabets and color names, and it is not clear how a similar explanation can be applied to the association between numerals and color names, this study suggests a potentially effective direction in the future study on the mechanisms of association between specific color names and specific graphemes. GPT-3 should be a useful tool to examine such possibility and may contribute to elucidate the mechanism of letter-color association in the future study.

In contrast to human non-synaesthetes, only a weak correlation was observed between the performance of the GPT-3 and grapheme-color synaesthetes. Synaesthetes associate letters and colors in specific ways that are different from those of non-synaesthetes, which should have resulted in low correlation. The procedure used in this study will be useful for estimating the answers of general people who share common knowledge with GPT-3. However, it will be difficult to apply this method to infer idiosyncratic responses that are based on specific traits or experiences, such as the graph–color association of synaesthetic subjects.

In the present study, we used the answer of GPT-3 for analysis only when it specified one of the 11 basic color terms. As we noted in Table 1 legend, overall frequency of answering basic color names by GPT-3 is less than one. Although a similar procedure was used in Simner et al. (2005), the overall frequency was nearly one. We think the difference in the overall frequency is caused by the difference in the control of the way the answer is given. In human subjects, task demand is easily understood and this would give strong control on the way the subjects make answers. On the other hand, in the present study, we made the question to GPT-3 as simple as possible. This necessarily sacrificed the context information given to GPT-3, and this may have yielded answers which cannot be included in the analysis. When we used the answers of GPT-3 that fit with the intended question, the frequency distributions of the basic color names were highly correlated with those of human non-synaesthetes. Because of this, we believe it is unlikely that the difference in the overall frequency between GPT-3 and human subjects is due to the difference in the color knowledge.

Although the test conducted in the present study is very simple, it shows the potential of AI systems with high language capability to be applied as a platform for studying how human perception is related to the knowledge and ability of language generation. AI systems that can generate natural language are still evolving and they will become useful tools for exploring the mechanisms of perception.

## Supplemental Material

sj-docx-1-ipe-10.1177_20416695221131832 - Supplemental material for Origin of the ease of association of color names: Comparison between humans and AI Click here for additional data file. Supplemental material, sj-docx-1-ipe-10.1177_20416695221131832 for Origin of the ease of association of color names: Comparison between humans and AI by Hidehiko Komatsu, Ami Maeno and Eiji Watanabe in i-Perception

sj-docx-2-ipe-10.1177_20416695221131832 - Supplemental material for Origin of the ease of association of color names: Comparison between humans and AI Click here for additional data file. Supplemental material, sj-docx-2-ipe-10.1177_20416695221131832 for Origin of the ease of association of color names: Comparison between humans and AI by Hidehiko Komatsu, Ami Maeno and Eiji Watanabe in i-Perception

sj-docx-3-ipe-10.1177_20416695221131832 - Supplemental material for Origin of the ease of association of color names: Comparison between humans and AI Click here for additional data file. Supplemental material, sj-docx-3-ipe-10.1177_20416695221131832 for Origin of the ease of association of color names: Comparison between humans and AI by Hidehiko Komatsu, Ami Maeno and Eiji Watanabe in i-Perception

sj-docx-4-ipe-10.1177_20416695221131832 - Supplemental material for Origin of the ease of association of color names: Comparison between humans and AI Click here for additional data file. Supplemental material, sj-docx-4-ipe-10.1177_20416695221131832 for Origin of the ease of association of color names: Comparison between humans and AI by Hidehiko Komatsu, Ami Maeno and Eiji Watanabe in i-Perception

sj-docx-5-ipe-10.1177_20416695221131832 - Supplemental material for Origin of the ease of association of color names: Comparison between humans and AI Click here for additional data file. Supplemental material, sj-docx-5-ipe-10.1177_20416695221131832 for Origin of the ease of association of color names: Comparison between humans and AI by Hidehiko Komatsu, Ami Maeno and Eiji Watanabe in i-Perception
