# Supplementary material for: Origin of the ease of association of color names: Comparison between humans and AI
Source: Iperception. 2022 Oct 26;13(5):20416695221131832. doi: 10.1177/20416695221131832 (PMC9623380; doi:10.1177/20416695221131832)
Supplement: sj-docx-1-ipe-10.1177_20416695221131832 - Supplemental material for Origin of the ease of association of color names: Comparison between humans and AI [file sj-docx-1-ipe-10.1177_20416695221131832.docx]

Supplemental material

Analysis of co-occurrence probability

We computed the co-occurrence probability between the alphabets and basic color names as well as between the numerals and basic color terms to examine if the co-occurrence probability will provide any information on the relationship between different types of graphemes and color names. We conducted Ngram analysis (bigram, trigram, 5gram, and 10gram) using token dataset from WikiText-103 (https://s3.amazonaws.com/research.metamind.io/wikitext/wikitext-103-v1.zip) that contains over 100 million tokens. Among the downloaded files, wiki.train.tokens (514 MB) was used for the analysis. The frequency of co-occurrence of words in the 11 basic color names and alphabets (or numerals) was counted by the nltk python library. The python code written for the analysis and the raw data obtained from the analysis are available on github (https://github.com/eijwat/bigram_ngram). We also counted the frequency of the 11 basic color names in the dataset (unigram, Table S2).

Supplemental Table S1 indicates the results of the Ngram analysis. We found that the co-occurrence probability was very similar for all cases tested. Table S3 summarizes the Pearson’s correlation coefficients across the log-transformed frequencies of basic color terms obtained by the Ngram analysis for numerals and alphabets shown in Table S1. As can be seen, correlation was quite high for most cases. Table S3 also shows the correlation coefficients between the results obtained by the Ngram analysis and the results of the unigram analysis (right-most column of Table S3). All the frequency distributions obtained by the Ngram analysis were significantly correlated with the frequency distribution obtained by the unigram analysis. This indicates that, in the present case, the co-occurrence probability does not provide meaningful information and simply reflects the frequency of the occurrence of the basic color names in the dataset. The results for the alphabets can be explained considering that each alphabet is an element of a word and individual alphabet rarely appears in the text except for special cases such as ‘a’ that is an indefinite article. Actually, the co-occurrence of ‘a’ and color terms are much higher than other alphabets. In the above-mentioned analysis, we counted the frequency distribution for alphabets in Ngram analysis both including and excluding the letter ‘a’, but the results did not significantly change. The results also suggest that numerals and color terms largely appear independently in the texts of dataset.

On the other hand, these co-occurrence probabilities were quite different from the frequency distribution of the basic color terms generated by GPT-3 in response to either the alphabets or the numerals shown in the main text. Supplemental Table S4 summarizes the Pearson’s correlation coefficients between the log-transformed frequencies of basic color terms obtained by the Ngram analysis for numerals and alphabets shown in Table S1 and the log-transformed frequencies of color terms generated by GPT-3 (Davinci engine, temperature=0.9) for alphabets and numerals. As can be seen, no significant correlation was observed between the frequency distributions obtained by any of the Ngram analysis or unigram analysis and those obtained by GPT-3. In contrast, there was high correlation between the frequency distributions obtained by GPT-3 for alphabets and for numerals (bottom of Table S4). These results support the idea that the results obtained by GPT-3 in the present study cannot be explained by simple co-occurrence of alphabet and color names in the trained data of GPT-3.
