# Supplementary material for: Origin of the ease of association of color names: Comparison between humans and AI
Source: Iperception. 2022 Oct 26;13(5):20416695221131832. doi: 10.1177/20416695221131832 (PMC9623380; doi:10.1177/20416695221131832)
Supplement: sj-docx-2-ipe-10.1177_20416695221131832 - Supplemental material for Origin of the ease of association of color names: Comparison between humans and AI [file sj-docx-2-ipe-10.1177_20416695221131832.docx]

Table S1

Results of the Ngram analysis for numerals and alphabets

In each of the Ngram analysis (bigram, trigram, 5gram or 10gram), we computed the number of cases of the co-occurrence between each grapheme (alphabet or numeral) and each of the basic color names. Total of the number of cases and the frequency relative to the total for each color name are shown at the right-most columns. For alphabet, the totals and the frequencies computed when ‘a’ is included and ‘a’ is excluded are both indicated.

------------------------------------------------------------------------------------------------

bigram numeral

0 1 2 3 4 5 6 7 8 9 total frequency

red 1 5 5 1 0 2 2 5 0 2 23 0.223

blue 0 3 1 1 0 1 0 0 0 0 6 0.058

green 0 2 2 2 0 1 0 0 0 0 7 0.068

yellow 0 0 1 2 0 1 0 0 0 0 4 0.039

orange 0 1 1 1 0 1 0 0 1 0 5 0.049

purple 0 0 0 0 0 1 0 0 0 0 1 0.010

pink 0 0 0 0 1 0 0 0 0 0 1 0.010

brown 2 2 0 1 4 1 0 0 1 0 11 0.107

white 0 2 8 1 2 2 2 0 1 1 19 0.184

black 4 3 5 7 1 1 2 0 0 2 25 0.243

grey 0 0 0 0 0 0 1 0 0 0 1 0.010

------------------------------------------------------------------------------------------------

bigram alphabet

a b c d e f g h i j k l m n o

red 1588 0 3 1 1 0 0 0 0 0 0 0 2 0 2

blue 846 1 1 0 0 0 0 0 1 0 0 0 0 15 1

green 625 0 0 1 1 0 1 2 1 0 0 0 1 0 1

yellow 557 0 3 0 0 0 1 0 1 0 0 0 0 0 0

orange 0 0 0 0 0 0 0 0 0 0 3 0 0 0 0

purple 153 0 0 0 0 0 0 0 0 0 0 0 0 0 0

pink 242 0 1 0 0 0 0 0 0 0 0 0 0 0 0

brown 223 0 0 0 0 0 0 2 4 0 0 0 0 1 0

white 2290 3 0 0 0 3 0 0 0 0 1 0 2 0 0

black 2353 0 0 0 1 1 0 0 2 0 0 1 0 11 0

grey 181 0 0 0 0 0 0 0 0 0 0 0 0 0 0

include 'a’ exclude 'a'

p q r s t u v w x y z total frequency total frequency

red 0 0 0 4 4 0 1 0 3 0 0 1609 0.174 21 0.114

blue 0 0 1 0 3 0 0 0 2 0 4 875 0.095 29 0.158

green 0 0 0 6 1 1 2 1 0 0 0 644 0.070 19 0.103

yellow 0 0 0 0 0 0 0 0 2 0 0 564 0.061 7 0.038

orange 0 0 0 0 1 0 0 0 0 0 0 4 0.000 4 0.022

purple 0 0 0 0 0 0 0 0 0 0 0 153 0.017 0 0.000

pink 0 0 0 0 1 0 0 0 0 0 0 244 0.026 2 0.011

brown 0 0 1 0 1 0 5 0 0 0 0 237 0.026 14 0.076

white 0 0 0 1 31 0 4 0 0 0 0 2335 0.253 45 0.245

black 1 0 0 5 12 0 1 2 1 0 0 2391 0.259 38 0.207

grey 0 0 0 4 0 0 1 0 0 0 0 186 0.020 5 0.027

------------------------------------------------------------------------------------------------

trigram numeral

0 1 2 3 4 5 6 7 8 9 total frequency

red 2 15 27 16 5 11 6 13 3 6 104 0.135

blue 2 12 9 2 1 4 3 0 1 2 36 0.047

green 1 20 15 14 6 10 2 1 2 6 77 0.100

yellow 0 6 6 4 0 3 3 0 0 0 22 0.029

orange 0 3 3 2 0 2 0 5 4 1 20 0.026

purple 0 0 0 0 0 2 0 0 0 0 2 0.003

pink 0 3 2 0 2 0 0 0 0 0 7 0.009

brown 5 16 2 8 14 6 3 1 5 1 61 0.079

white 38 40 42 33 24 26 18 20 15 15 271 0.352

black 20 21 38 25 11 11 18 5 5 8 162 0.210

grey 1 1 0 1 2 0 2 1 0 0 8 0.010

------------------------------------------------------------------------------------------------

trigram alphabet

a b c d e f g h i j k l m n o

red 3762 1 13 2 3 0 0 0 8 1 1 0 7 0 7

blue 2120 3 3 2 0 0 2 3 7 0 0 1 3 33 2

green 1638 3 0 3 6 0 3 4 7 0 0 0 2 0 3

yellow 1355 1 6 0 0 0 2 0 4 2 0 1 2 0 1

orange 191 0 0 0 0 0 0 0 4 0 7 1 1 0 0

purple 350 0 0 0 0 0 0 0 0 0 1 0 0 0 0

pink 607 1 2 0 0 0 0 0 2 0 0 0 0 0 1

brown 1108 2 0 18 0 0 1 4 13 0 0 0 6 3 3

white 5578 7 5 4 4 6 0 3 14 0 2 2 7 1 2

black 5572 4 2 1 5 2 0 0 16 1 0 2 6 33 2

grey 536 0 0 0 0 0 0 0 2 0 0 0 1 0 0

include 'a’ exclude 'a'

p q r s t u v w x y z total frequency total frequency

red 1 0 1 12 8 0 3 4 16 1 0 3851 0.164 89 0.128

blue 0 0 2 0 7 0 3 0 4 0 8 2203 0.094 83 0.119

green 0 0 0 16 2 2 5 2 1 0 0 1697 0.072 59 0.085

yellow 2 0 2 2 0 0 1 0 4 0 0 1385 0.059 30 0.043

orange 0 0 0 0 2 0 0 0 0 0 0 206 0.009 15 0.021

purple 0 0 0 1 0 0 1 1 0 0 0 354 0.015 4 0.006

pink 1 1 0 0 5 0 1 0 0 0 0 621 0.026 14 0.020

brown 0 0 3 11 5 0 10 0 0 0 0 1187 0.050 79 0.113

white 1 2 1 7 65 0 13 7 0 2 0 5733 0.244 155 0.222

black 2 0 0 15 25 0 4 5 10 0 3 5710 0.243 138 0.198

grey 0 0 0 26 1 0 2 0 0 0 0 568 0.024 32 0.046

------------------------------------------------------------------------------------------------

5gram numeral

0 1 2 3 4 5 6 7 8 9 total frequency

red 22 104 145 87 67 75 47 59 35 30 671 0.141

blue 19 55 66 55 23 15 30 13 14 13 303 0.064

green 23 115 70 72 44 38 29 17 36 36 480 0.101

yellow 3 42 40 20 11 14 24 12 7 4 177 0.037

orange 0 20 14 15 8 12 10 22 9 5 115 0.024

purple 1 1 6 2 2 6 5 0 0 2 25 0.005

pink 3 14 17 13 9 6 1 1 3 0 67 0.014

brown 25 88 38 43 55 30 45 18 38 18 398 0.084

white 179 249 191 189 149 157 102 110 77 99 1502 0.316

black 114 149 158 127 91 88 85 44 52 56 964 0.203

grey 4 11 2 6 12 4 8 5 0 4 56 0.012

------------------------------------------------------------------------------------------------

5gram alphabet

a b c d e f g h i j k l m n o

red 10502 11 41 14 12 4 5 6 69 3 6 0 40 3 25

blue 6156 22 7 13 6 5 6 18 46 0 4 8 16 69 16

green 5082 15 8 15 18 0 8 14 55 2 3 3 16 0 16

yellow 3697 6 15 6 4 6 9 0 33 6 0 7 15 0 5

orange 976 0 4 4 0 1 0 0 17 0 19 3 7 1 0

purple 975 0 2 0 0 0 0 0 5 0 3 0 2 0 0

pink 1754 4 4 0 0 2 2 0 7 0 0 0 2 2 10

brown 4759 23 4 74 2 0 3 10 63 1 0 0 35 9 29

white 16045 30 23 21 20 14 5 12 116 3 4 13 48 7 16

black 15512 25 11 18 16 7 9 0 132 7 0 10 34 78 21

grey 1781 0 1 1 0 0 0 0 20 0 0 0 6 0 4

include 'a’ exclude 'a'

p q r s t u v w x y z total frequency total frequency

red 5 2 6 34 30 2 19 13 55 5 1 10913 0.155 411 0.133

blue 1 0 6 17 20 0 9 5 10 5 18 6483 0.092 327 0.106

green 0 0 0 65 11 4 15 6 5 1 0 5362 0.076 280 0.091

yellow 4 0 6 20 3 2 5 2 14 0 1 3866 0.055 169 0.055

orange 1 0 1 3 5 0 2 0 2 0 0 1046 0.015 70 0.023

purple 0 1 0 3 0 0 4 3 2 0 0 1000 0.014 25 0.008

pink 3 3 0 2 18 2 3 0 0 2 0 1820 0.026 66 0.021

brown 0 1 14 41 13 0 24 0 0 0 2 5107 0.073 348 0.113

white 6 7 7 58 141 0 37 23 8 11 6 16681 0.237 636 0.206

black 16 3 8 51 70 1 23 14 64 6 11 16147 0.230 635 0.206

grey 0 0 0 79 3 0 7 0 0 0 1 1903 0.027 122 0.039

-----

------------------------------------------------------------------------------------------------

10gram numeral

0 1 2 3 4 5 6 7 8 9 total frequency

red 289 1033 1023 840 606 640 435 393 304 254 5817 0.158

blue 150 484 488 440 315 181 258 223 163 119 2821 0.077

green 270 736 551 446 406 242 243 170 216 186 3466 0.094

yellow 132 289 346 246 159 207 145 136 82 89 1831 0.050

orange 34 161 143 161 99 115 93 120 101 62 1089 0.030

purple 14 26 60 26 43 59 23 17 13 8 289 0.008

pink 28 94 111 121 70 47 28 33 38 8 578 0.016

brown 253 614 406 398 463 359 404 208 286 239 3630 0.099

white 938 1635 1250 1186 979 1060 689 738 619 671 9765 0.266

black 699 1050 917 922 691 649 474 384 375 380 6541 0.178

grey 51 99 117 190 100 57 88 56 50 71 879 0.024

-------------------------------------------------------------------------------------------

10gram alphabet

a b c d e f g h i j k l m n o

red 38300 143 157 99 113 37 40 40 533 8 43 42 241 45 143

blue 22449 169 53 77 27 38 25 81 366 4 30 41 152 193 161

green 20262 109 75 67 78 18 34 60 411 14 21 38 129 3 95

yellow 12524 38 65 33 31 24 36 0 242 20 4 25 83 3 25

orange 5274 11 29 26 4 6 0 0 103 5 63 15 27 10 4

purple 3300 12 7 2 12 0 0 0 103 0 8 0 10 2 9

pink 6161 27 12 10 14 9 14 1 97 5 4 0 23 12 79

brown 22277 157 57 238 17 4 17 28 556 15 0 0 172 27 174

white 57901 196 156 123 114 53 69 67 836 13 42 71 324 67 140

black 54985 167 75 176 112 51 43 19 1111 44 14 64 267 239 160

grey 7499 0 12 7 6 0 0 0 140 0 1 2 47 0 44

include 'a’ exclude 'a'

p q r s t u v w x y z total frequency total frequency

red 24 23 46 217 134 25 112 45 228 29 10 40877 0.152 2577 0.138

blue 17 0 27 148 110 11 86 25 56 37 52 24435 0.091 1986 0.106

green 11 2 25 247 67 14 60 24 56 16 15 21951 0.081 1689 0.090

yellow 9 0 22 110 27 15 25 12 58 0 6 13437 0.050 913 0.049

orange 6 0 7 19 28 4 17 4 14 1 0 5677 0.021 403 0.022

purple 0 7 0 10 12 0 28 8 9 1 5 3545 0.013 245 0.013

pink 8 12 12 12 82 21 9 2 21 7 0 6654 0.025 493 0.026

brown 0 10 115 185 101 6 65 4 38 5 16 24284 0.090 2007 0.108

white 51 26 60 339 412 4 172 108 131 43 37 61555 0.228 3654 0.196

black 60 27 79 269 311 42 125 61 425 38 75 59039 0.219 4054 0.217

grey 0 1 0 255 11 2 27 10 43 15 22 8144 0.030 645 0.035
