# Supplementary material for: Origin of the ease of association of color names: Comparison between humans and AI
Source: Iperception. 2022 Oct 26;13(5):20416695221131832. doi: 10.1177/20416695221131832 (PMC9623380; doi:10.1177/20416695221131832)
Supplement: sj-docx-3-ipe-10.1177_20416695221131832 - Supplemental material for Origin of the ease of association of color names: Comparison between humans and AI [file sj-docx-3-ipe-10.1177_20416695221131832.docx]

Table S2

Results of the unigram analysis

number of

cases hit frequency

red 20618 0.152

blue 11372 0.084

green 11839 0.087

yellow 5890 0.043

orange 3579 0.026

purple 1507 0.011

pink 2845 0.021

brown 14276 0.105

white 28725 0.212

black 27232 0.201

grey 7665 0.057
