# Supplementary material for: Origin of the ease of association of color names: Comparison between humans and AI
Source: Iperception. 2022 Oct 26;13(5):20416695221131832. doi: 10.1177/20416695221131832 (PMC9623380; doi:10.1177/20416695221131832)
Supplement: sj-docx-4-ipe-10.1177_20416695221131832 - Supplemental material for Origin of the ease of association of color names: Comparison between humans and AI [file sj-docx-4-ipe-10.1177_20416695221131832.docx]

Table S3

Peasons's correlation coefficients across the log-transformed frequencies of basic color terms obtained by the Ngram analysis for numerals and alphabets (shown in Table S1) and unigram analysis (shown in Table S2). For alphabets, both frequencies including 'a' and those not including 'a' are examined.

For log-transformation, we added the same constant value (minimum number of non-zero value 0.000433 that was for orange in bigram for alphabet inclluding 'a') to avoid the presence of zero value (1 case: purple for bigram excluding 'a').

bigram trigram 5gram 10gram bigram trigram 5gram 10gram

<-------------numeral------------> <-----alphabet (include ‘a’)----->

------------------------------------------------------------------------------------------------------------------

bigram numeral 0.946 0.946 0.941 0.533 0.789 0.861 0.900

*** *** *** ** *** ***

------------------------------------------------------------------------------------------------------------------

trigram numeral 0.946 0.994 0.989 0.608 0.845 0.913 0.951

*** *** *** * ** *** ***

------------------------------------------------------------------------------------------------------------------

5gram numeral 0.946 0.994 0.990 0.670 0.885 0.941 0.968

*** *** *** * *** *** ***

------------------------------------------------------------------------------------------------------------------

10gram numeral 0.941 0.989 0.990 0.672 0.885 0.945 0.975

*** *** *** ** *** *** ***

------------------------------------------------------------------------------------------------------------------

bigram alphabet 0.533 0.608 0.670 0.672 0.931 0.867 0.799

(include 'a') * * * *** *** **

------------------------------------------------------------------------------------------------------------------

trigram alphabet 0.789 0.845 0.885 0.885 0.931 0.987 0.957

(include 'a') ** ** *** *** *** *** ***

------------------------------------------------------------------------------------------------------------------

5gram alphabet 0.861 0.913 0.941 0.945 0.867 0.987 0.991

(include 'a') *** *** *** *** *** *** ***

------------------------------------------------------------------------------------------------------------------

10gram alphabet 0.900 0.951 0.968 0.975 0.799 0.957 0.991

(include 'a') *** *** *** *** *** *** ***

------------------------------------------------------------------------------------------------------------------

bigram alphabet 0.787 0.903 0.889 0.914 0.551 0.768 0.836 0.879

(exclude 'a') ** *** *** *** ** ** ***

------------------------------------------------------------------------------------------------------------------

trigram alphabet 0.839 0.930 0.931 0.959 0.698 0.877 0.932 0.963

(exclude 'a') ** *** *** *** * *** *** ***

------------------------------------------------------------------------------------------------------------------

5gram alphabet 0.871 0.946 0.952 0.975 0.730 0.905 0.955 0.980

(exclude 'a') *** *** *** *** * *** *** ***

------------------------------------------------------------------------------------------------------------------

10gram alphabet 0.883 0.941 0.958 0.969 0.786 0.941 0.980 0.994

(exclude 'a') *** *** *** *** ** *** *** ***

------------------------------------------------------------------------------------------------------------------

unigram 0.865 0.938 0.935 0.965 0.712 0.889 0.941 0.971

*** *** *** *** * *** *** ***

bigram trigram 5gram 10gram unigram

<-----alphabet (exclude ‘a’)----->

----------------------------------------------------------------------------------

bigram numeral 0.787 0.839 0.871 0.883 0.865

** ** *** *** ***

----------------------------------------------------------------------------------

trigram numeral 0.903 0.930 0.946 0.941 0.938

*** *** *** *** ***

----------------------------------------------------------------------------------

5gram numeral 0.889 0.931 0.952 0.958 0.935

*** *** *** *** ***

----------------------------------------------------------------------------------

10gram numeral 0.914 0.959 0.975 0.969 0.965

*** *** *** *** ***

----------------------------------------------------------------------------------

bigram alphabet 0.551 0.698 0.730 0.786 0.712

(include 'a') * * ** *

----------------------------------------------------------------------------------

trigram alphabet 0.768 0.877 0.905 0.941 0.889

(include 'a') ** *** *** *** ***

----------------------------------------------------------------------------------

5gram alphabet 0.836 0.932 0.955 0.980 0.941

(include 'a') ** *** *** *** ***

----------------------------------------------------------------------------------

10gram alphabet 0.879 0.963 0.980 0.994 0.971

(include 'a') *** *** *** *** ***

----------------------------------------------------------------------------------

bigram alphabet 0.957 0.943 0.898 0.913

(exclude 'a') *** *** *** ***

----------------------------------------------------------------------------------

trigram alphabet 0.957 0.994 0.977 0.985

(exclude 'a') *** *** *** ***

----------------------------------------------------------------------------------

5gram alphabet 0.943 0.994 0.989 0.984

(exclude 'a') *** *** *** ***

----------------------------------------------------------------------------------

10gram alphabet 0.898 0.977 0.989 0.975

(exclude 'a') *** *** *** ***

----------------------------------------------------------------------------------

unigram 0.913 0.985 0.984 0.975

*** *** *** ***

* p<0.05, ** p<0.01, *** p<0.001
