# Supplementary material for: Origin of the ease of association of color names: Comparison between humans and AI
Source: Iperception. 2022 Oct 26;13(5):20416695221131832. doi: 10.1177/20416695221131832 (PMC9623380; doi:10.1177/20416695221131832)
Supplement: sj-docx-5-ipe-10.1177_20416695221131832 - Supplemental material for Origin of the ease of association of color names: Comparison between humans and AI [file sj-docx-5-ipe-10.1177_20416695221131832.docx]

Table S4

Peason's correlation coefficients between the log-transformed frequencies of basic color terms

obtained by the Ngram analysis and those obtained by GPT-3 for alphabets and numerals (Davinci engine, temperature=0.9).

For log-transformation, we added the same constant value (minimum number of non-zero value 0.000433 that was for orange in bigram for alphabet inclluding 'a') to avoid the presence of zero value (1 case: purple for bigram excluding 'a').

Correlation coefficients between the frequencies obtained by GPT-3 for alphabets and numerals are also shown at the bottom.

GPT-3

alphabet numeral

bigram numeral 0.180 0.293

trigram numeral 0.091 0.164

5gram numeral 0.130 0.193

10gram numeral 0.100 0.164

bigram alphabet (include 'a') 0.116 0.118

trigram alphabet (include 'a') 0.133 0.180

5gram alphabet (include 'a') 0.092 0.147

10gram alphabet (include 'a') 0.059 0.122

bigram alphabet (exclude 'a') 0.091 0.112

trigram alphabet (exclude 'a') -0.018 0.023

5gram alphabet (exclude 'a') 0.019 0.062

10gram alphabet (exclude 'a') 0.035 0.086

unigram -0.059 0.015

GPT-3 alphabet vs numeral 0.963***

* p<0.05, ** p<0.01, *** p<0.001
